# Rare-earth-doped TiO_2_ rutile as a promising ferromagnetic alloy for visible light absorption in solar cells: first principle insights

**DOI:** 10.1039/d0ra05725h

**Published:** 2020-09-25

**Authors:** A. Fakhim Lamrani

**Affiliations:** Nanomaterial and Nanotechnology Unit, E. N. S. Rabat, Energy Research Center, Faculty of Sciences, Mohammed V University B.P. 1014 Rabat Morocco fakhim@um5.ac.ma

## Abstract

The electronic structure and magneto-optic properties of TiO_2_ (rutile) doped with two concentrations of rare-earth (RE) elements are explored using a first-principle all-electron full-potential augmented spherical-wave method based on the PBEsol–GGA approximation, to examine their potential use as a spintronic and optoelectronic system. The results predict that all compounds exhibit half-metallic character, the only exception is by doping with Nd or that the material is magnetic but the cloud is still a half-metallic magnet. We also found that the localized level at the Fermi energy shifts to lower energy as the atomic number of the 4f-element increases. Consequently, the mechanism that controls the ferromagnetism in these systems has been proposed according to this positioning. The energy of the localized level due to Gd is sufficiently low to lie at the top of the valence band, while Eu produces a midgap state. However, the Fermi level was not noticed precisely at the middle of the energy gap. In contrast, the impurity states of the Nd-, Pm-, and Sm-dopants are close to the bottom of the conduction band of the host system. This allows electrons to be delocalized, and gives a higher scattering cross-section. Interestingly, the analysis of optical absorption and electrical conductivity emphasizes that this ferromagnetic DMS based on rare-earth elements has the power to be a promising spintronic device for visible light absorption in solar cells. Finally, the relationship between the mechanism that controls the ferromagnetism and the absorption efficiency of visible light is discussed.

## Abstract 2

The electronic structure and magneto-optic properties of TiO_2_ (rutile) doped with rare-earth elements are explored using a first-principles all-electron full-potential augmented spherical-wave method, to examine their potential use as a spintronic and optoelectronic system.

## Graphical abstract

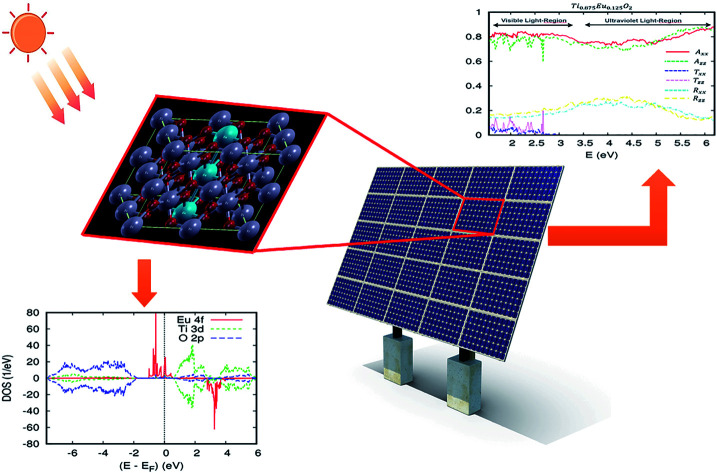

## 1 Introduction

Titanium dioxide (TiO_2_) has an enormous functionality and has been spread over a wide range of application domains, including photocatalysts,^1^ pigments, and sensors.^2^ Recently, TiO_2_ has also offered potential applications as a mother compound of electronic materials. It seems that when they are doped with transition metals (TMs), this material develops some unlooked-for physical properties, such as ferromagnetic alloys for magneto-optical devices^3^ and transparent conducting oxide (TCO).^4^ However, these properties could have important consequences in the developing technology. Despite these promising properties, the undoped and TM-doped TiO_2_ materials remain active only under ultraviolet (UV) light and their effective utilization of visible light is one of the important challenges to increase their utility in a photovoltaic cell.

Not long ago, the rare-earth-based dilute magnetic semiconductor (DMS) has drawn much attention owing to many applications in photoelectric devices and optical communication fields. Meanwhile, TiO_2_ is regarded as a good candidate host for rare-earth doping owing to its good properties (*e.g.*, mechanical, thermal, and anticorrosive properties).^5^ Some prominent results were discovered in research studies on rare-earth-doped TiO_2_ phosphor.^6–11^ For instance, TiO_2_ : Eu^3+^ has a strong quality emission of Eu^3+^ in the red-light region. The emission peak wavelength and the shape of the emission line, in addition to the line width, are insensitive to variation of temperatures from 12 to 300 K.^11^ These features make TiO_2_ : Eu^3+^ a very interesting material for technological applications. It has been reported that the TiO_2_ : Tb^3+^ and TiO_2_ : Eu^3+^ gel films deposited on porous anodic alumina by the sol–gel method have great amelioration of the luminescence intensity compared with Si as the substrate.^12^

Notwithstanding the efforts realized by several research groups, the area of the spintronic coupling with optoelectronics is almost intact. There is seldom a theoretical and experimental study on this subject, or more specifically, the making of solar cells based on diluted ferromagnetic semiconductors, which may be a key material for the future technology of solar cells. Indeed, the ferromagnetic materials may display the enhanced lifetimes of excited states owing to the spin-dependent transition selection rules, which are not yet considered. For a photovoltaic energy conversion system, a long lifetime of excited states is important, as they ameliorate the performance and give the opportunity so that the photogenerated carriers will be collected. In this context, the main problem with the photovoltaic solar cells was the conversion efficiency limitation until now.^13–17^ This is because of the discord between the solar incident spectrum and the spectral absorption of the cell's material.

Various methods have been developed, which consist of modifying the solar spectrum by a wavelength conversion process in such a way as to render the high-efficiency photovoltaic conversion. Among the emerging ideas are the promising up and down-conversion approaches that are realized by doping the parent material with rare-earth elements.^18,19^

Since the electronic structure and magneto-optic properties of doped TiO_2_ are susceptible to the experimental conditions, it is necessary to investigate from the theoretical calculation. Within this framework, the pragmatic GGA approach works reasonably well and shows a better agreement with the experimental data compared to the LDA + *U* calculations.^20^

The aim of this paper is twofold: (a) to supply a more accurate and complementary study on the electronic structure, and to describe the magnetic ground-state of the TiO_2_ rutile doped with rare-earth elements using first-principles calculations based on PBEsol–GGA; (b) to determine the rendering of these ferromagnetic DMS under natural light, which is predominantly in the visible region, and its ability to photovoltaic conversion in solar cells. Finally, the relationship between the mechanism responsible for the ferromagnetism in rare-earth-doped TiO_2_ and the absorption efficiency under visible light is discussed.

## 2 Method of calculations

The rutile TiO_2_ crystallizes in a tetragonal cell (*a* = 4.587 Å, *c* = 2.954 Å, space group *P*4_2_/*mnm*). In this study, we used a 2 × 2 × 2 supercell (16 Ti and 32 O atoms) and replaced the Ti atoms with one and two RE atoms, corresponding to concentration ratios of *x* = 1/16, and 2/16 in Ti_1−*x*_RE_*x*_O_2_, which are close to the experimentally reported values.^21^ The ferromagnetic stability was determined by the total energy difference (Δ*E*) of the supercell between the ferromagnetic (FM) state and antiferromagnetic (AFM) state. The calculations were based on density functional theory^22,23^ using the generalized gradient approximation (GGA–PBEsol) with the Perdew, Ruzsinszky, Csonka *et al.* (PBEsol) approximation,^24,25^ and the local-density approximation parameterized according to Perdew and Wang, PW.^26^ The calculations were performed using the scalar-relativistic implementation of the full-potential augmented spherical wave (FP-ASW) method^27–30^ based on the atomic sphere approximation (ASA). In this method, the wave functions are expanded in atom-centered augmented spherical waves, which are the Hankel functions and numerical solutions of Schrödinger's equation, respectively, outside and inside the so-called augmentation spheres. To optimize the basis set, additional augmented spherical waves were placed at carefully selected interstitial sites. The choice of these sites, as well as the augmentation radii, was automatically determined using the sphere-geometry optimization SGO algorithm.^31^ Self-consistency was achieved by a highly efficient algorithm for convergence acceleration.^32^ The Brillouin zone (BZ) integrations were performed with an increasing number of *k*-points (7 × 7 × 10) in order to ensure convergence of the results with respect to the space grid. The geometry was fully relaxed using the Hellman–Feynman force and total energy. The convergence criterion was fixed to 10^−8^ Ry in the self-consistent procedure and charge difference Δ*Q* = 10^−8^ between two successive iterations.

## 3 Results and discussion

### 3.1 Electronic properties

**Fig. 1:**
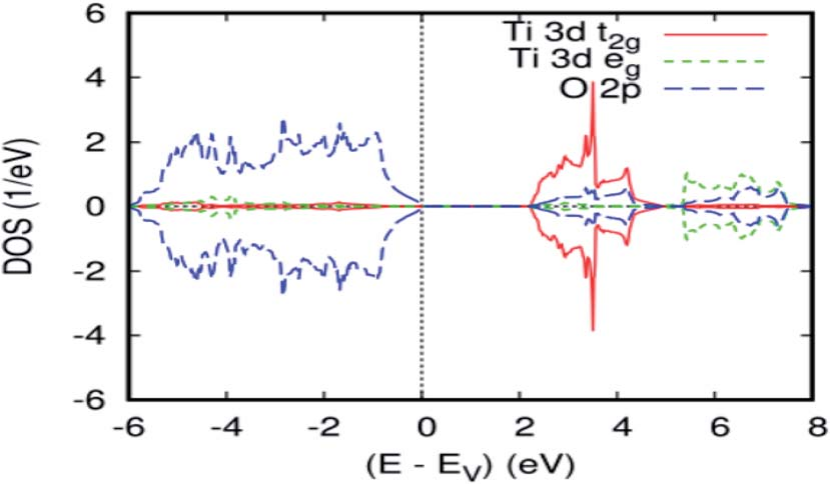
The PBEsol projected local DOS (PLDOS) for dioxide titan.

In order to verify the consistency of our results, we studied the electronic properties of the parent material (TiO_2_). The overall band structure of the present FP-ASW result is consistent with the existing results.^33–37^Fig. 1 presents the projected local DOS (PLDOS) for titanium dioxide. It can be seen that the majority and minority spin channels are symmetrical. This signifies that the TiO_2_ rutile is a nonmagnetic material. Indeed, the valence band is chiefly dominated by O-2p states and is full, whereas the conduction band is formed principally by Ti-3d and is empty. The latter splits into t_2g_ and e_g_ sub-bands by the octahedral crystal field. The calculated bandwidth for the valence band is 6.00 eV, which is in agreement with the experimental data.^38^ As usual in the PBEsol–GGA calculations, the obtained energy gap of 2.2 eV is underestimated. In fact, this shortcoming is well known and accounted for by employing the GGA.^39^ The optimized bulk cell parameters for pure TiO_2_ with FPASW–GGA^40^ are in agreement with the experimental data^41,42^ and other theoretical values.^43^

### 3.2 Electronic and magnetic properties

After mentioning the structural and electronic properties of the parent material TiO_2_ (rutile), we will now investigate the spin-resolved electronic and magnetic properties of the substitutional rare-earth (RE) impurities in TiO_2_ (rutile). The two concentrations that we considered are 6.25 and 12.5%. Since there are two possible configurations of spin ions (ferromagnetic (FM) and antiferromagnetic (AFM) alignments), we studied both couplings for all RE-doped TiO_2_ materials. The obtained results are presented in Table 2, and indicate that the lower energy state favors the parallel-spin alignment.

Generally, for an oxide semiconductor (in particular TiO_2_) to become ferromagnetic after being doped with 3d elements, it is necessary to create oxygen vacancies in the host matrix.^44^ In contrast, our results predicted that we do not need vacancies to obtain a suitable property in spintronic devices in the case of the doped TiO_2_ rutile with rare-earth elements. Moreover, the projected local DOS for all dopings of TiO_2_ with 4f-elements ensures that both majority- and minority-spins of the rare-earth elements are located in a bandgap of the host matrix. This indicates that the introduction of rare-earth impurities does not destroy the semiconducting nature of the titanium oxide materials. Besides, a robust half-metallic nature is a favorable feature with 100% spin polarization in the ground state, and large half-metallic gaps of about 2.3 eV were obtained for these compounds. Another plus is that the total magnetic moments of these alloys are sufficiently large. The combination of these aspects for titanium oxide doped with rare-earth elements is encouraging for fabricating spintronic devices.

To explain the magnetic ground–state properties of the rare-earth elements-doped TiO_2_ rutile, we calculated the spin-polarized electronic structure for both concentrations at 6.25% and 12.5%. The obtained results revealed that the majority-spins due to the 4f rare-earth elements shift to lower energies as the atomic number of the 4f-ions increase. According to the positioning of the relative energy to the dopant in the bandgap of the parent material, and based on the projected local DOS of Ti_1−*x*_(RE)_*x*_O_2_, we distinguished between the three and separated them into groups. Therefore, the energy of the localized level due to Gd is sufficiently low to lie at the top of the valence band. Fig. 3a and b show the total density of states (DOS) of GGA–PBEsol and the rare earth 4f projected local DOS (PLDOS) for Ti_0.875_Gd_0.125_O_2_, respectively. We found that some of the valence bands cross the Fermi level. Hence, the 100% polarization appears on the majority-spins of the Gd-4f ions, with one resulting in a half-metallic nature for this material. The calculation of the half-metallic gap for both concentrations is illustrated in Tables 1 and 2. The result indicated that it is almost the same, and does not change with increasing Gd concentration.

**Table 1:** Total energy *E*_t_, electrical conductivity *σ* (Ω m)^−1^, energy gap *E*_g_, moment total *m*_t_ and partial *m*_O_, *m*_RE-4f_ for Ti_0.9375_RE_0.0625_O_2_

Compounds	Ti_1−*x*_Nd_*x*_O_2_	Ti_1−*x*_Pm_*x*_O_2_	Ti_1−*x*_Sm_*x*_O_2_	Ti_1−*x*_Eu_*x*_O_2_	Ti_1−*x*_Gd_*x*_O_2_
*m*_RE-4f_ (*μ*_B_)	1.482176	2.784277	3.954044	5.017542	5.989344
*m*_O_ (*μ*_B_)	−0.018011	−0.027816	−0.020330	−0.04760	−0.048673
*m*_t_ (*μ*_B_)	2000	3000	4.000	5000	6000
*E*_g_ (eV)	2.33	2.26	2.2	2.2	2.3
*σ* (Ω m)^−1^	8.7 × 10^4^	1.4 × 10^4^	1.76 × 10^4^	3.9 × 10^4^	2.5 × 10^4^
*E*_t_ (Ryd) (FM)	−49 632.692201	−50 425.786376	−51 238.830566	−52 072.388289	−52 926.847628

**Table 2:** Total energy *E*_t_, electrical conductivity *σ* (Ω m)^−1^, energy gap *E*_g_, moment total *m*_t_ and partial *m*_O_, *m*_RE-4f_ for Ti_0.875_RE_0.125_O_2_

Compounds	Ti_1−*x*_Nd_*x*_O_2_	Ti_1−*x*_Pm_*x*_O_2_	Ti_1−*x*_Sm_*x*_O_2_	Ti_1−*x*_Eu_*x*_O_2_	Ti_1−*x*_Gd_*x*_O_2_
*m*_RE-4f_ (*μ*_B_)	1.6	2.881408	3.976863	5.040104	6.046040
*m*_O_ (*μ*_B_)	−0.029864	−0.042308	−0.058221	−0.0820	−0.095000
*m*_t_ (*μ*_B_)	4.000	6.000	8000	10 000	12 000
*E*_g_ (eV)	2.286	2.4	2.4	2.36	2.3
*σ* (Ω m)^−1^	1.57 × 10^5^	4.5 × 10^4^	2.2 × 10^4^	7.4 × 10^4^	6.0 × 10^4^
*E*_t_ (Ryd) (FM)	−67 168.422921	−68 755.220937	−70 381.186858	−72 048.134089	−73 756.651946
*E*_t_ (Ryd) (AFM)	−67 168.400668	−68 755.191040	−70 381.170139	−72 048.106012	−73 756.644883

**Fig. 2:**
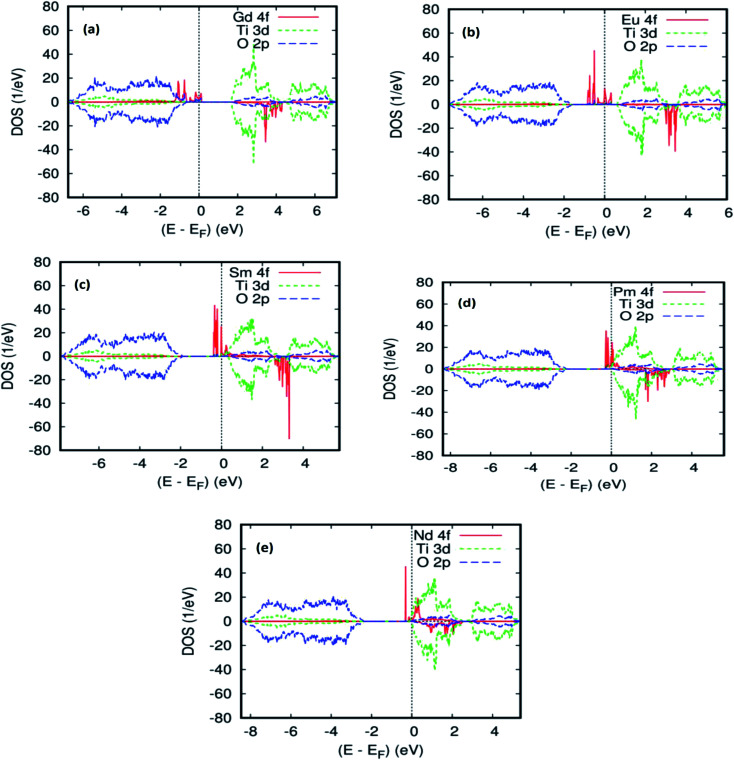
(a) Projected local DOS for Ti0.9375Gd0.0625O2, (b) projected local DOS for Ti0.9375Eu0.0625O2, (c) projected local DOS for Ti0.9375Sm0.0625O2, (d) projected local DOS for Ti0.9375Pm0.0625O2, (e) projected local DOS for Ti0.9375Nd0.0625O2. The Fermi level is set at zero.

**Fig. 3:**
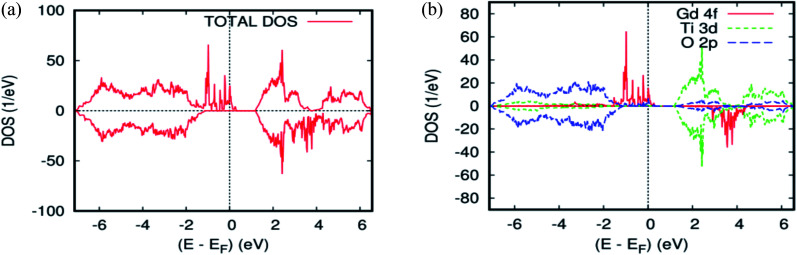
The PBEsol projected local DOS for Ti_0.875_Gd_0.125_O_2_. (a) The total DOS; (b) the partial DOS of the total Ti-3d, total O-2p, and Gd-4f states. The Fermi level is set at zero.

To elucidate the origin of the various bands and half–metallic properties for Ti_1−*x*_Gd_*x*_O_2_, (*x* = 0.0625 and 0.125), the spin-polarized atomic partial density of states is presented in Fig. 2a and 3b. As seen from this figure, the states between −6.8 eV and −1 eV primarily consisted of O-2p with a small contribution from the Ti-3d states. The spin-up channel of the Gd-4f impurity states and the Fermi level are close to the top of the valence band, and the polarization at the Fermi energy increased with increasing Gd concentration. However, in the case of the spin-down channel, these Gd-4f states are located between t_2g_ and e_g_ of the host system Ti-3d, leading to a wide energy bandgap. The difference between the two spin channels is due to the large exchange splitting of the Gd-4f states. According to the descriptions above, the origin of the half–metallic properties of Ti_1−*x*_Gd_*x*_O_2_ (*x* = 0.0625 and 12.5), a careful analysis of the spin density reveals that the majority-spin channel of Gd-4f overlaps with that of O-2p at the Fermi level *E*_f_. These characters indicate a strong hybridization between Gd and its neighboring O atoms. Therefore, it is the p–f exchange mechanism that is responsible for ferromagnetism in the Gd-doped TiO_2_.

The total and local magnetic moments in the ground-state structure of the Gd-doped TiO_2_ rutile are offered in Table 2. The integer value of the total magnetization in the unit cell is consistent with the half-metallic characteristic of this compound. The main part of this magnetic moment is strongly localized on the Gd site with a magnetic moment of 6.04 *μ*_B_. The nearest neighbor host atoms are weakly polarized with induced moments of +0.04 *μ*_B_ on the nearest neighbor Ti sites and −0.0095 *μ*_B_ on the nearest neighbor O site between the two Gd atoms, which indicate that the nearest neighbor O-2p electrons prefer the AFM alignment to Gd-4f.

**Fig. 4:**
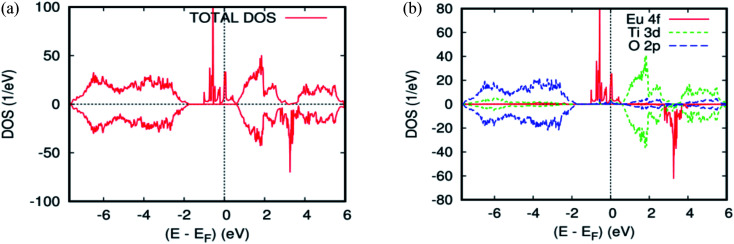
The PBEsol projected local DOS for Ti_0.875_Eu_0.125_O_2_. (a) The total DOS; (b) the partial DOS of the total Ti-3d, total O-2p, and Eu-4f states. The Fermi level is set at zero.

Fig. 2b and 4a, b show the GGA–PBEsol calculated total density of states (DOS) and the Eu-4f projected density of states (PDOS). For Ti_1−*x*_Eu_*x*_O_2_ (*x* = 0.0625 and 0.125), the Eu-based system is half-metallic (*i.e.*, spin-up channel (-down channel) are polarized (unpolarized) at the Fermi level, *E*_f_). The main features obtained for the pure rutile TiO_2_ were also obtained for Ti_1−*x*_Eu_*x*_O_2_: the upper VB region comes from the O-2p orbitals, while the lower CB region has mainly Ti-3d characters. On the other hand, a new series of Eu-4f states were observed in the bandgap region, which indicates that the introduction of europium impurities in the TiO_2_ matrix does not have any effect on the semiconducting nature of the parent material.

From Fig. 2b and 4b, three groups of energy levels for Eu-4f in the spin-up states can be observed. The midgap states just below the Fermi level are filled. The energy level is polarized at the Fermi level. The states above the Fermi level are located near the minimum of the CB and are empty, while the spin-down-induced states are located between the triply degenerate t_2g_ state and doubly generate e_g_ state. As is clear from Tables 1 and 2, the Eu-doped TiO_2_ showed the largest magnetic moment of 5 Bohr magneton, *μ*_B_. This is from the seven valence electrons, two for making a band with oxygen and five for a magnetic moment. Each nearest neighboring O-2p atom connecting to Eu has a magnetization of −0.0476 *μ*_B_ for the single substitutional impurity and −0.082 *μ*_B_ for the two substitutional impurities, which indicates that the nearest neighbor O-2p electrons preferred the (AFM) alignment over that of Eu-4f. According to our calculations, either of the density of states or the magnetic moment, there is no sign of charge transfer between the two europium atoms in the host matrix. This is because they have the same oxidation state, suggesting that the double-exchange mechanism will not be efficacious for the observed ferromagnetism in Ti_14_Eu_2_O_32_. As a matter of fact, the europium produces midgap states, but the Fermi level was not exactly observed in the middle of the energy gap. This impedes the motion of the charge carriers from the impurity states to the conduction band. Consequently, the lowest energy was the one in which the magnetic impurities were located closest to each other, suggesting localization of the impurities. The Eu 4f PLDOS's in the doped case look very similar to those of the Cr/Mn codoping TiO_2_ rutile.^45^ From these data, another process of exchange mechanism must be invoked. Thus, the Fermi level of the diluted magnetic semiconductor almost is in the middle of the bandgap, and the carriers are localized around the donor ions. This ferromagnetic behavior may be attributed to the presence of magnetic polarons due to the localized carriers. It is well known that the effective radius of the polaron depends on the temperature and concentration of donor ions.^46,47^ If the density of the polarons and their radii are large enough to exceed the threshold of percolation, the magnetic interaction will be ferromagnetic.

**Fig. 5:**
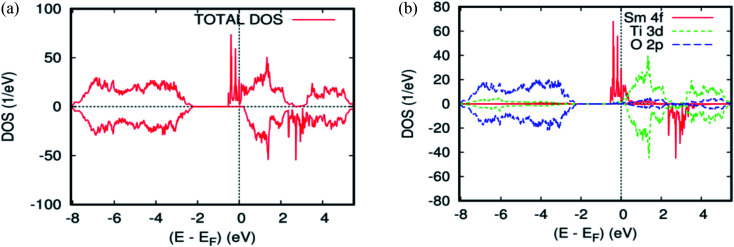
The PBEsol projected local DOS for Ti_0.875_Sm_0.125_O_2_. (a) The total DOS; (b) the partial DOS of the total Ti-3d, total O-2p, and Sm-4f states. The Fermi level is set at zero.

**Fig. 6:**
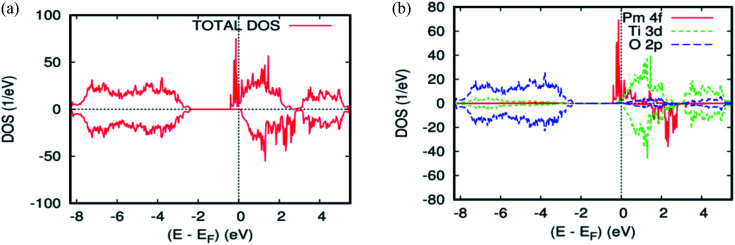
The PBEsol projected local for Ti_0.875_Pm_0.125_O_2_. (a) The total DOS; (b) the partial DOS of the total Ti-3d, total O-2p, and Pm-4f states. The Fermi level is set at zero.

**Fig. 7:**
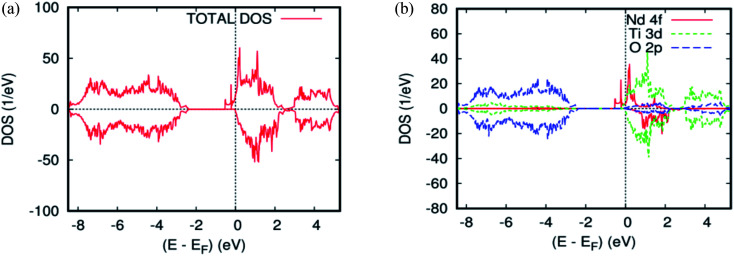
The PBEsol projected local for Ti_0.875_Nd_0.125_O_2_. (a) The total DOS; (b) the partial DOS of the total Ti-3d, total O-2p, and Nd-4f states. The Fermi level is set at zero.

As far as the three rare-earth-based systems, Sm (Fig. 2c and 5b), Pm (Fig. 2d and 6b), and Nd (Fig. 2e and 7b) are concerned, we observed that the spin-down states for all three rare earth materials are mostly unoccupied, following a trend common to most DMS.^48^ On the other hand, a difference between Sm, Pm, and Nd in the exchange splitting has been noted. The approximate energy position of these states decreased in energy as the atomic number decreased. Moreover, we expect an even stronger hybridization with t_2g_ of a host system. For the spin-up states, the projected local DOS (PLDOS) of the Sm- and Pm-doping showed the half-metallic nature of the electronic structures, in which the conduction electrons at the Fermi level *E*_f_ are 100% spin-polarized. In fact, in the case of Nd-doping, the material is a magnetic metal, but it can still be a half-metallic magnet. The majority, spin 4f states of Sm, Pm, and Nd seems to be hybridized strongly with the conduction band (Fig. 5a, 6a, and 7a), so that the impurity states and the Fermi level are close to the bottom of the conduction band of the parent material (generally Ti-3d). Therefore, the production of the spintronic systems, in which the impurity states are lying close to CBM, is of significant interest to improving the performance of these systems. This allows for the delocalization of the electrons, and gives a higher cross-section scattering.

Due to the overlapping of the impurity states with the conduction band in Sm-, Pm-, and Nd-doped TiO_2_ materials, the underlying mechanism responsible for the ferromagnetism is the same as that mentioned in the study of the doped ZnO material with double impurities, Zn_1−2*x*_Fe_*x*_Co_*x*_O^49^ and Mo-doped TiO_2_ rutile.^50^ In fact, the oxidation states of the Sm-, Pm-, and Nd-, impurities in the host matrix are measured roughly from the carrier's occupancy in the 4f orbitals. Tables 1 and 2 show the PBEsol-calculated total and partial magnetic moments for both rare-earths concentration (*x* = 0.625 and 0.125) for the FM spin configurations. Furthermore, the oxidation states of the two impurity ions in Ti_14_(RE)_2_O_2_ are the same. It means that there is no indication of Zener's double exchange (absence of charge transfer) or p–f hybridization mechanism. In this case, one needs to invoke another exchange mechanism between the rare-earth materials, according to the so-called RKKY-type exchange interaction mediated by Ti 4s carriers or conduction carriers induced by oxygen vacancies. In fact, all of the most important criteria for fabricating spintronic devices, like the free flow of charge carriers, and the half-metallic nature, were achieved.

### 3.3 Optical properties

**Fig. 8:**
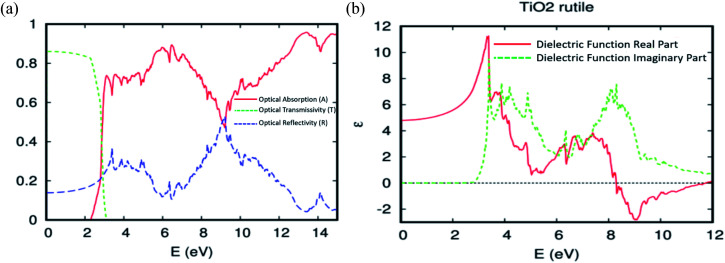
The (a) optical absorption, transmissivity, and reflectivity. (b) The real and imaginary parts of the dielectric function for the TiO_2_ rutile.

According to the fundamental relationship between optical performance, electronic structures, and electrical conductivity, we can understand the behaviour of the ferromagnetic DMS based on rare-earth elements under natural light, and its ability to photovoltaic conversion in the solar cell. First, we checked the optical absorption, transmissivity, reflectivity, and dielectric function for the TiO_2_ rutile without doped elements. As usual, the absorption in the visible light region depends on the calculated bandgap, as shown in Fig. 8a. However, the pure TiO_2_ rutile had no response to the visible light and only responded to light from *hν* > 2.3 eV. Indeed, the parent material transmits in this area at almost 86% of the natural light. To analyze the optical properties in more detail, the real and imaginary parts of the dielectric function *ε* = *ε*_1_ + i*ε*_2_*vs.* photon energy *hν* = 0–12 eV, were plotted in Fig. 8b. The imaginary part *ε*_2_ curve is zero below the onset of direct interband transitions. It increases steeply, is nearly linearly from *hν* > 2.9 eV to reach *ε*_2_ = 9.5 at 3.4 eV, and then decreases to 5.0 at *hν* = 3.6 eV. The effective utilization of visible light by titanium dioxide is one of the important challenges for its increased utility as a photovoltaic conversion device. So, to make it active under natural light, it is required to dope the hot matrix with rare-earth elements.

**Fig. 9:**
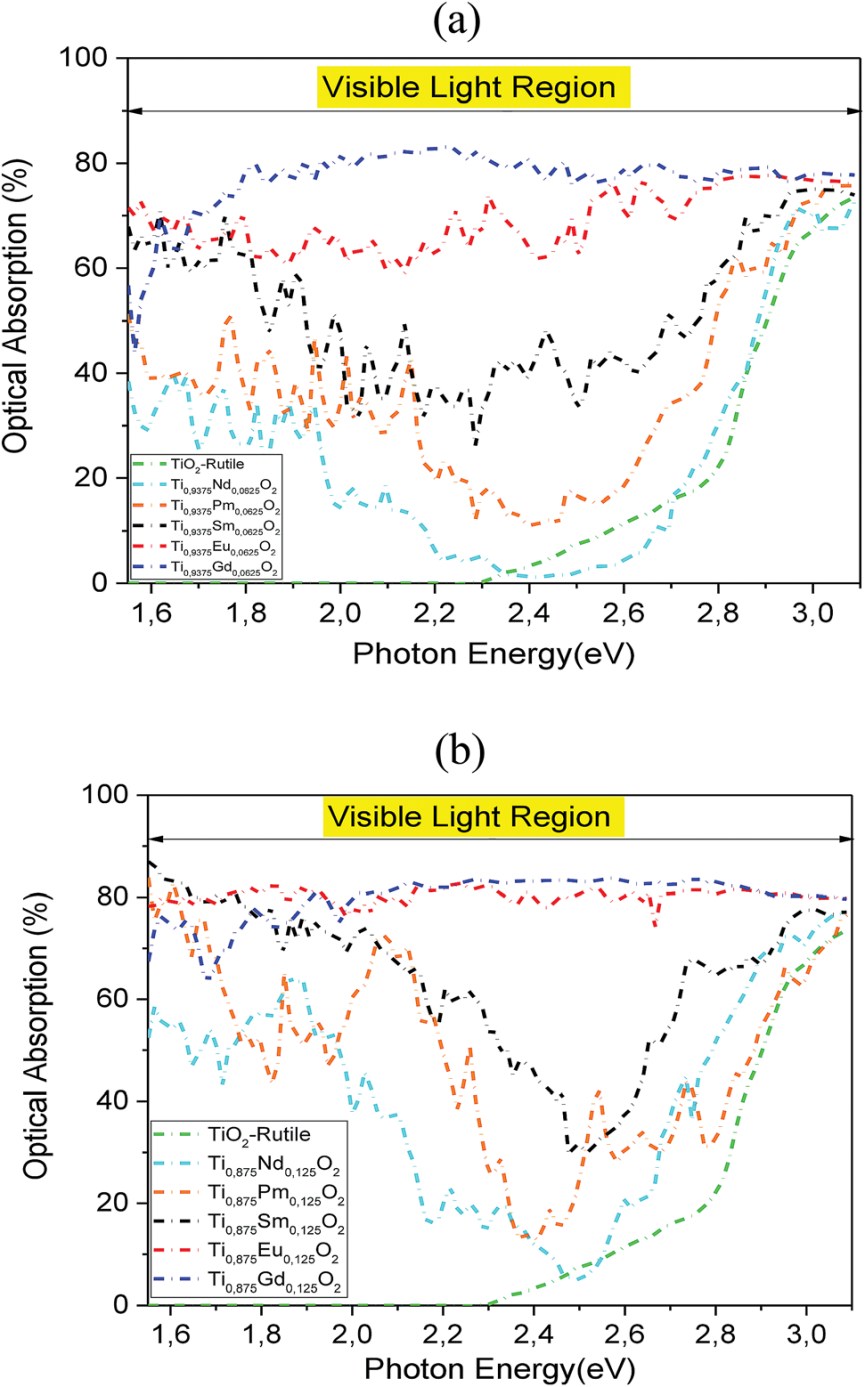
(a) The optical absorption in visible light for TiO_2_, Ti_0.9375_Nd_0.0625_O_2_, Ti_0.9375_Pm_0.0625_O_2_, Ti_0.9375_Sm_0.0625_O_2_, Ti_0.9375_Eu_0.0625_O_2_, and Ti_0.9375_Gd_0.0625_O_2_ with the fixed slab thickness of *d* = 1000 nm. (b) The optical absorption in visible light for TiO_2_, Ti_0.875_Nd_0.125_O_2_, Ti_0.875_Pm_0.125_O_2_, Ti_0.875_Sm_0.125_O_2_, Ti_0.875_Eu_0.125_O_2_, and Ti_0.875_Gd_0.125_O_2_ with the fixed slab thickness of *d* = 1000 nm.

To investigate the influence of 4f-elements on the optical properties, we calculated the optical absorption in visible light for the Nd-, Pm-, Sm-, Eu- and Gd-doped TiO_2_ at two concentrations and compared them with those for the pure TiO_2_. As plotted in Fig. 9a and b, it is clear that the insertion of rare-earth elements by substitution in the TiO_2_ matrix completely changed its optical properties under natural light. The comparison of the obtained results with that of the parent material ensures that the Sm-, Eu-, and Gd-doped systems display an excellent optical absorption, particularly in the visible light region, due to the hybridized 4f-states that appear in the bandgap region. On the contrary, the Nd and Pm dopings fail to exhibit excellent visible light absorption in the wavelength range that is equivalent to *hν* = 2.2–2.7 eV. Furthermore, we noticed that the optical absorption in the visible region was enhanced with increasing atomic number and concentration of the 4f elements. As a matter of fact, the highly strong absorption was achieved when we doped the parent material by europium and gadolinium ions, with a bit of superiority in favor of Ti_0.875_Eu_0.125_O_2_ in the photonic region, *hν* = 1.5–2.0 eV. For *hν* > 2.0 eV, the two absorption curves have almost the same behavior under natural light. It is clear that the insertion of Eu and Gd by substitution in the hot matrix of the TiO_2_ rutile allowed us to achieve a good correspondence between the incident solar spectrum and positioning of the 4f states in a bandgap. Indeed, Ti_0.875_Eu_0.125_O_2_ absorbed in the range of 75–84% of solar photons, while Ti_0.875_Gd_0.125_O_2_ absorbed in the range of 64–84%.

**Fig. 10:**
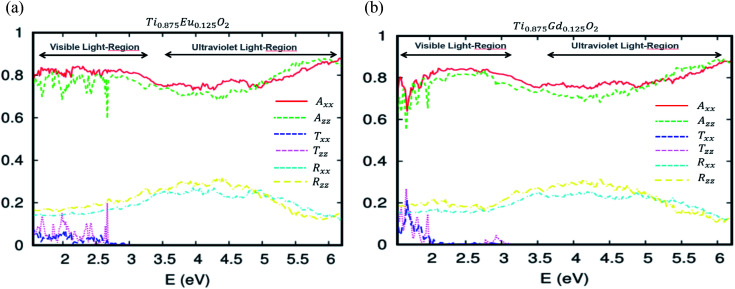
The optical absorption, transmissivity and reflectivity in visible light for (a) Ti_10.875_Eu_0.125_O_2_ and (b) Ti_10.875_Gd_0.125_O_2_. The fixed slab thickness is *d* = 1000 nm.

More detail on the optical behavior of the Eu- and Gd-doped TiO_2_ is presented in Fig. 10a and b, respectively. These curves describe how the optical absorption, transmissivity, and reflectivity under visible and ultraviolet light can be different, depending on the crystallographic directions. The absorption study states that both A_*xx*_ and A_*zz*_ are roughly isotropic. Therefore, the absorption is uniform in all orientations. Above all, it is clear that the transmittance in the visible region is quasi-negligible. On the other side of the optical absorption, it can reach 84%. The optical reflectivity is correctly defined by the rule 1 = *R* + *A* + *T*, where *R* is the optical reflectivity. For *A* and *T*, they are defined as the optical absorption coefficient and optical transmission coefficient, respectively. This high efficiency of the photonic conversion leads us to think that these ferromagnetic alloy-based 4f-elements convert more than the visible domain photons. The up-conversion and down-conversion has also been achieved. Within this framework of the down-conversion, we found that this DSM based on europium and gadolinium can also absorb in the ultraviolet light region (UV) in the range of 72–90%. In effect, the high absorption of the UV photons leads to a very efficient photonic conversion process; therefore, the quantum cutting is realized.

Often, when we insert rare-earth species in a single or double way inside a matrix, the hot quantum cutting of the high energy photons has to be achieved.^51,52^ So, for the photon conversion process to be efficient, it requires a high absorption of UV photons, which is the case of the europium- and gadolinium-doped TiO_2_ (rutile). Furthermore, this couple already has shown great capability in this context when inserted into a LiGdF_4_ matrix.^53^

**Fig. 11:**
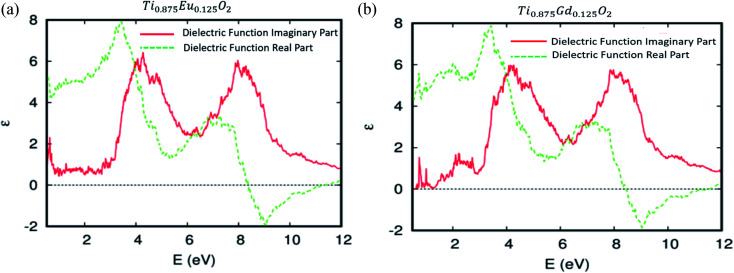
The real and imaginary parts of the dielectric function *versus* energy for (a) Ti_10.875_Eu_0.125_O_2_ and (b) Ti_10.875_Gd_0.125_O_2_.

The predicted dielectric functions of Ti_0.875_Eu_0.125_O_2_ and Ti_0.875_Gd_0.125_O_2_ are offered in Fig. 11a and b, respectively. We concentrate on the imaginary part, which describes the absorption of the electromagnetic waves attributable to the interband transitions. One can see sharper peaks in the region of lower energy photons and the photons associated with the visible domain. It should be noted that in the case of the parent material, the curve is zero in this area. This is attributed to some hybridized energy states in the bandgap of the parent material, and also to the competitive role of the 4f-states associated with the Eu and Gd ions in the decrease of the photon excitation energy. As a consequence, the Eu-, and Gd-4f states could be an effective link of free flow charge carriers between the valence band edge and the bottom of the conduction band under natural light.

**Fig. 12:**
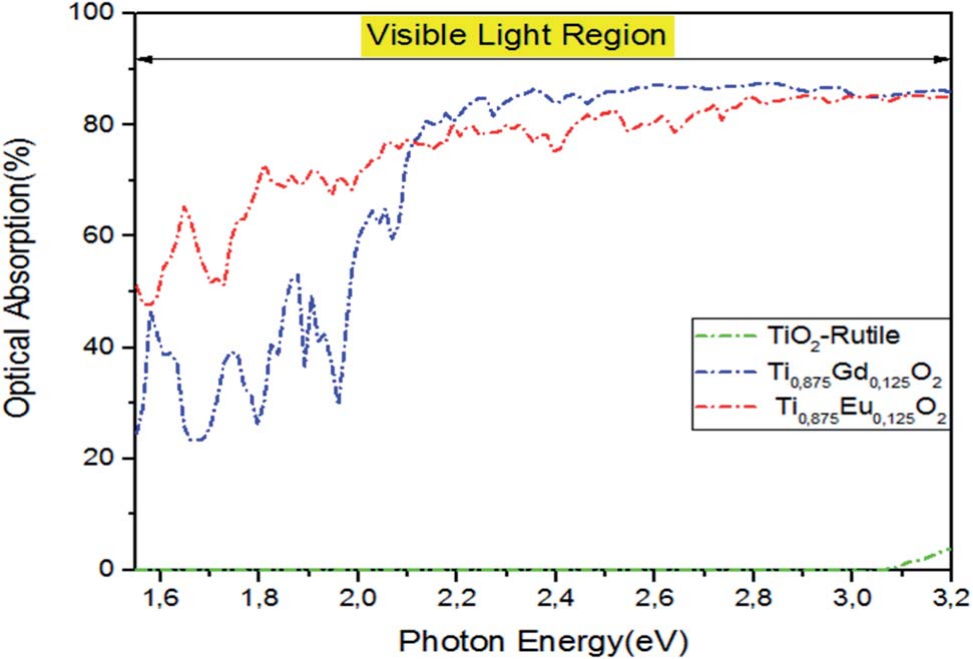
The PBEsol + *U* optical absorption in visible light for pure TiO_2_, Ti_10.875_Gd_0.125_O_2_, and Ti_10.875_Eu_0.125_O_2_. The fixed slab thickness is *d* = 1000 nm.

A special interest is placed upon the effect of optical absorption of Ti_14_EuO_32_ and Ti_14_GdO_32_ under natural light if we had produced an experimental bandgap. The pragmatic PBEsol + *U* approach was employed.^54^ In addition, double-counting corrections were included within the fully localized limit (FLL).^55^ We applied the Hubbard coefficient only on the Ti-3d states of the host system. The single-shot PBEsol–GGA + *U*_Ti_ functional raises the gap value at 3.0 eV with *U* = 4.4 eV, which is very favorable for the experimental investigation. As illustrated in Fig. 12, the three plotted optical absorption spectra in the range of 1.5–3.2 eV showed that the parent material had no response to the visible light. It only responds to light from *hν* > 3.2 eV. Indeed, the optical absorption curve in the interval photon energies of *hν* = 1.55–2.1 eV fluctuates between 50% and 76% in the case of Ti_0.875_Eu_0.125_dO_2_. In the case of Ti_0.875_Gd_0.125_O_2_, the curve fluctuates between 27% and 68%. From *hν* > 2.1 eV, both curves behave like the one achieved by the PBEsol–GGA functional.

**Fig. 13:**
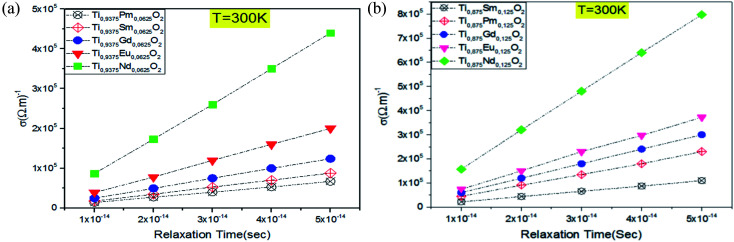
Electrical conductivity as a function of the relaxation time at *T* = 300 K: (a) for Ti_0.9375_RE_0.0625_O_2_, (b) for Ti_0.875_RE_0.125_O_2_.

Given the above results, releasing electrons by the absorption of visible light is highly desirable to gain a high power photovoltaic conversion, but it is not enough. For this reason, it is necessary to know how well Ti_0.875_Eu_0.125_O_2_ and Ti_0.875_Gd_0.125_O_2_ can let pass and conduct the electric current. The transport property calculations for both concentrations ensured an excellent electrical conductivity for these materials. Fig. 13a and b clarify a linear relationship between the electrical conductivity and relaxation time for both concentrations. In fact, when the relaxation time was assumed to be 10^−14^ s, the value of the electrical conductivity at 300 K was on the order of 10^4^ (Ω m)^−1^ (see Tables 1 and 2). The charge carriers' mobility is high enough, and the material is in the range of the conductors. The combinations of these aspects for the TiO_2_ rutile doped with Eu and Gd allow us to predict that the material will feasibly be of great power to a highly effective photovoltaic conversion in solar cells, and maybe a key for the future technology of solar cells.

In addition, this study gives us an idea about identifying an approach on the relationship between the underlying mechanism responsible for the ferromagnetism and the absorption efficiency of rare-earth-doped TiO_2_ in visible light. Since we have a linear relationship between energy and frequency, we consider that a beam of the incident light is made up of a flux of photons whose energy is determined by the frequency of visible light. Indeed, as long as the frequency of the light wave *υ*_visible_ is lower than that of the threshold frequency *υ*_0_, which is linked to the position of the 4f states in the bandgap, some released electrons by the photovoltaic effect do not arrive at the bottom of the band conduction. So, in the case of the p–f hybridization or the band magnetic polaron mechanism, some hybridized energies lie at the top of the valence band and in the midgap states. This will allow us to realize the double cut and decrease the photon excitation energy, which in turn can lead to red-shift benefitting from most of the visible-light photons. Consequently, the good correspondence between the incident solar spectrum and the spectral absorption of the ferromagnetic alloys was obtained. As a matter of fact, the hybridized energies of the 4f-states that appear in the bandgap could be an effective link of the free flow charge carriers between the valence band edge and the bottom of the conduction band under natural light. All of this could result in a high effectiveness of the photovoltaic conversion, which is like the case of Eu, Gd-doped TiO_2_ rutile. When the hybridized energies of the 4f-states appeared close to the bottom of the conduction band of the parent material, an RKKY-type exchange interaction was produced. The excitation energy of the photons became great. Therefore, *υ*_visible_ ≤ *υ*_0_, and this explains the low rendering of Nd and Pm in the range of *hν* = 1.55–2.7 eV.

## 4 Conclusion

In this piece of work, we systematically investigated the electronic structure and magneto-optic properties of rare-earth-doped TiO_2_ rutile using first-principles insights. The half-metallic behavior for Ti_1−*x*_(RE)_*x*_O_2_ (*x* = 0.065 and 0.125) was achieved and is energetically stable. The mechanism that controls the ferromagnetism in these systems has been proposed according to the hybridized energy of 4f-states in the bandgap. So, the results also indicate that in the case of the p–f hybridization or the band magnetic polaron mechanism, the ferromagnetic DMS based on the rare-earth elements has the power to be a promising spintronic device for visible light absorption in solar cells. Another plus is that the GGA–PBEsol calculation guarantees excellent electrical conductivity. The combination of these aspects for titanium oxide doped with rare-earth is very important for fabricating spintronic devices operating in the wavelength range of 400–800 nm.

## Conflicts of interest

There are no conflicts to declare.

## Supplementary Material
